# Supplementary material for: High-quality de novo genome assembly for the Galápagos endemic lava gull using Oxford Nanopore Technologies
Source: G3 (Bethesda). 2025 Dec 19;16(3):jkaf259. doi: 10.1093/g3journal/jkaf259 (PMC12958806; doi:10.1093/g3journal/jkaf259)
Supplement: jkaf259_Supplementary_Data [file jkaf259_supplementary_data.docx]

**Supplementary Material**

**The Generation of a High-Quality *De Novo* Genome Assembly for the Galápagos Endemic Lava Gull Using Oxford Nanopore Technologies**

Jessica A. Martin, James B. Henderson, Vera de Ferran, Gabriela Pozo, Alice Skehel,

Athena Lam, John P. Dumbacher, Jaime A. Chaves

**Sequencing Methods and Preparation**

Sample preparation and sequencing occurred at the GSC. Due to the isolation of the archipelago, we brought in all the consumables (various sized tubes, pipette tips, gloves, nuclease free water, and Qubit tubes and reagents), kits (Monarch HMW, ONT Ulta-Long DNA Sequencing Kit, and ONT Wash Kit), and equipment (sequencing laptop, P2 Solo Sequencer, and flow cells) needed for the wet lab work. The GSC supplied Phosphate Buffer Saline, 98% Ethanol, and Isopropanol.

The NEB UHMW Extraction protocol recommended a starting input of 5ul for nucleated blood; however, because our samples were stored in roughly a 1:2 ratio of blood to NAP buffer, we began with a starting input of 20ul. Another modification made to our protocol was during the first part of the extraction, where the protocol calls for the sample to be centrifuged at 4℃, which we were unable to do as the centrifuge at the GSC did not have a temperature setting, however, this did not affect the extraction gDNA yield from our sample. We were also extremely gentle with our genome sample and did not vortex the sample during the extraction and library preparation steps. Instead, whenever the protocol called for the sample to be vortexed, we would slowly invert the tube four to five times. For both the genome extraction and the library preparation, we used regular pipette tips instead of the wide-bore tips outlined in the protocol.

Additionally, we experienced some technical difficulties with our sequencing laptop and P2 Solo sequencer. While sequencing the genome sample, every couple of hours, the sequencer would appear to be sequencing on the computer in the MinKNOW GUI, with the lights flashing correctly and GUI saying it was still sequencing; however, in the system messages, we would get a script error requiring us to force stop the “sequencing” on the computer. After reaching out to ONT, they informed us that there are existing issues with using the Thunderbolt 4 port that can lead to run instability, and they recommended that the P2 Solo should not be connected to the computer with the ONT supplied USB-C to USB-C cable; instead, it needed to be connected with an ONT supplied USB-C to USB-B cable, with the USB-C end connected to the P2 Solo and the USB-B end connected to the computer. While sequencing in the field, we did not have the recommended ONT USB-C to USB-B cable, but we did have a non-ONT brand USB-C to USB-B cable from a SanDisk Extreme PRO with USB4 - 4TB (Model Number: SDSSDE82-4T00-G25). Switching to the USB-C to USB-B cable fixed our issue with the sequencing abruptly stopping partway through runs.

The lava gull genome was sequenced using two flow cells, PAY93342 and PAY93362, purchased in June of 2024, less than a month before our field season. After the initial flow cell check, PAY93342 and PAY93362 had 6729 pores and 5236 pores, respectively. The flow cells were each loaded once with 90 μL (1.143μg) of library. The library was sequenced across a total of seven runs, five of which were not completed, primarily due to repeated crashing issues that required us to restart the runs each time they occurred. As a result, we chose not to wash and reload the flow cell, but instead to restart it after each crash and continue until the pores were depleted. The only runs we were able to stop manually were the first, which was ended 2 hours and 40 minutes after initiation to move the machine to a surge-protected outlet, and the third, which was stopped after 6 hours and 10 minutes prior to reloading onto a new flow cell. The other five runs terminated due to script errors: two crashed after approximately 5 hours and 20 minutes, while the remaining three failed in under an hour and a half (Flowcell Info Table Below). Because these crashes prevented the software from generating the standard run report, run and flow cell information reported here was determined from the pore scan .csv file output. Despite the sequencing issues we encountered, we were able to generate 1,251,699 pass reads from flow cell PAY93342 and 717,156 pass reads from flow cell PAY93362. This was calculated from the sequencing_summary.txt file with the command:

**tail -n +2 sequencing_summary.txt | cut -f1 | sed 's/_.*//' | sort | uniq -c**

**Flowcell Info** - Initial sequencing number of runs, length of run time, initial pores in flow cell run, and end of the run. Blue rows indicate which runs were crashed, and white rows indicate which runs we manually stopped.

| Run Name | Flow Cell | Run Time | Run Status | Pores First Scan | Pores Last Scan |
| --- | --- | --- | --- | --- | --- |
| LGSCZ01UHMW_R1 | PAY93342 | 2hrs 40min | Stopped | 6729 | 5994 |
| LASCZ02UHMW_R2 | PAY93342 | 5hrs 20min (approx) | Crashed | 5069 | 2891 |
| LG_SCZ02_UHMW_R3 | PAY93342 | 6hrs 10min | Stopped | 3057 | 1072 |
| LaGu_SCz02_UHMW_R4 | PAY93362 | 5hrs 20min (approx) | Crashed | 5236 | 3049 |
| LaGu_SCz02_UHMW_R5 | PAY93362 | Less than 1hr 30min | Crashed | 2187 | Only 1 scan recorded |
| LaGu_SCz02_UHMW_R6 | PAY93362 | Less than 1hr 30min | Crashed | 1930 | Only 1 scan recorded |
| LaGu_SCz02_UHMW_R7 | PAY93362 | Less than 1hr 30min | Crashed | 1577 | Only 1 scan recorded |

**Supplemental Tables and Figures**

**Table S1** - Initial sequencing generated basecalled reads made up of 1.78 million reads, comprising 29.5 Gbp bases, a mean read length of 16.6 kbp, and a mean read quality of 17.8.

| **Initial Sequencing Statistics for the Lava Gull Genome** | |
| --- | --- |
| Total Bases Generated | 29.6 Gbp |
| Number of Reads | 1.78 million |
| Mean Read Length | 16,585 bases |
| Mean Read Quality | 17.8 |
| Coverage | 22.5x |

**Table S2** - Initial sequencing results, including reads and bases generated, mean read length and coverage, from lava gull and four other bird species using various sequencing platforms.

| Metric | lava gull | Galápagos petrel | black-headed gull | little vermillion flycatcher | dark-eyed junco |
| --- | --- | --- | --- | --- | --- |
| Sequencing Method | ONT Ultra-Long Reads (1 library) | ONT Ultra-Long Reads (2 libraries) | PacBio HiFi | PacBio HiFi  and Hi-C | Chicago and Dovetail Hi-C |
| Reads Generated | 1.78 million | 4.10 million | 5.08 million | 6.84 million | 218 million and 121 million |
| Bases Generated | 29.5 Gbp | 49.1 Gbp | 51.3 Gbp | 789 Gbp | 121 Gbp |
| Mean Read Length | 16.5 kbp | 11.9 kbp | N/A | 12.5 kbp | N/A |
| Coverage | 22.5x | 36.07x | 37x | 79x (with Hi-C) | 117x |

**Table S3** - Sequencing statistics after the adaptor removal with Porechop.

Coverage estimation is calculated using 1.32 Gbp as the genome size.

N50 31554 L50 301103. mean 16480.57. median 9854.

N10 66935 L10 35464

N20 53779 L20 84856

N30 45041 L30 144771

N40 37882 L40 216031

N50 31554 L50 301103

N60 25712 L60 404266

N70 20083 L70 533426

N80 14440 L80 705230

N90 8361 L90 968218

N100 148 L100 1784432

mode Q23 230,531 4.34G

Q10+ 1,782,176 29.40G 99.97% 22.27X

Q12+ 1,730,061 28.54G 97.04% 21.62X

Q15+ 1,633,904 26.93G 91.58% 20.40X

Q20+ 1,293,095 21.98G 74.76% 16.65X

Q25+ 340,997 5.59G 19.00% 4.23X

Q30+ 8,926 29.6M 0.10% 0.02X

1+ 1,784,432 29.41G 100.00% 22.28X

1000+ 1,750,804 29.38G 99.90% 22.26X

5000+ 1,190,542 27.93G 94.96% 21.16X

10000+ 885,318 25.71G 87.42% 19.48X

15000+ 685,610 23.24G 79.02% 17.60X

30000+ 325,804 15.46G 52.58% 11.72X

50000+ 107,419 7.05G 23.98% 5.34X

100000+ 4,045 515.1M 1.75% 0.39X

22.28X coverage for 1.32 Gbgenome. 1,784,432 recs. 29,408,453,302 bases.

1,784,432 reads. 29,408,453,302 bases. 21.19 Q score mean.

**Genome Assembly**

In addition to the assembly used to generate the final genome, Flye assemblies were generated using both of the differently trimmed reads, as well as assembled with HiFiasm v0.24 [(Cheng et al., 2021)](https://www.zotero.org/google-docs/?lYbfKs) using the non-corrected, porechop-trimmed reads. However, all other generated assemblies were missing more than one BUSCO and not as complete as the Flye assembly with non-corrected Porechop trimmed reads.

**Flye assembly statistics**

**Table S4** - Assembly statistics from the flye assembly

genome_size=1.32g reads=lavagull_filt_primary.fastq

flye.sh --nano-hq $reads --out-dir . --scaffold -g $genome_size

flye_assembly.stats

Number of scaffolds 1300

Total size of scaffolds 1346675606 1.347G

Longest scaffold 140686371 140.686M

Shortest scaffold 520

Number of scaffolds > 1M nt 75 5.8%

Number of scaffolds > 10M nt 28 2.2%

N50 scaffold length 38356818 L50 scaffold count 10 38.357M

N90 scaffold length 1421244 L90 scaffold count 60 1.421M

Number of contigs > 1M nt 85 6.2%

Number of contigs > 10M nt 30 2.2%

N50 contig length 37118489 L50 contig count 12 37.118M

N90 contig length 1339042 L90 contig count 74 1.339M

C:8334[S:8317,D:17],F:3,M:1,n:8338

C:99.95%[S:99.75%,D:0.20%],F:0.04%,M:0.01%,n:8338

**HiFiasm assembly statistics**

**Table S5**  - Assembly statistics from the HiFiasm assembly

telo=CCCTAA

adapter_trimmed_ONT_reads=LavaGull_primary.fastq

hifiasm.sh -t $threads --telo-m $telo --ont $adapter_trimmed_ONT_reads

hifiasm.asm.bp.p_ctg.stats

Number of contigs 671

Total size of contigs 1302602735 1.303G

Longest contig 120210382 120.21M

Shortest contig 9119

Number of contigs > 1M nt 88 13.1%

Number of contigs > 10M nt 24 3.6%

auN50 49964914 49.965M

N50 contig length 41169231 L50 contig count 9 41.169M

N90 contig length 1396874 L90 contig count 73 1.397M

C:8316[S:8299,D:17],F:7,M:15,n:8338

C:99.74%[S:99.53%,D:0.20%],F:0.08%,M:0.18%,n:8338

**Scaffolded assembly statistics**

**Table S6** - Assembly statistics from the scaffolded flye assembly

---------------- Information for assembly 'assembly_v0.9.fasta' ----------------

Number of scaffolds 1187

Total size of scaffolds 1348411092

Longest scaffold 218127570

Shortest scaffold 520

Number of scaffolds > 1K nt 1168 98.4%

Number of scaffolds > 10K nt 957 80.6%

Number of scaffolds > 100K nt 344 29.0%

Number of scaffolds > 1M nt 42 3.5%

Number of scaffolds > 10M nt 18 1.5%

Mean scaffold size 1135982

Median scaffold size 42793

N50 scaffold length 84761895 L50 scaffold count 5

N60 scaffold length 72296394 L60 scaffold count 7

N70 scaffold length 55825834 L70 scaffold count 9

N80 scaffold length 22577350 L80 scaffold count 13

N90 scaffold length 3465058 L90 scaffold count 26

scaffold %A 27.61 number of A 372340251

scaffold %C 22.39 number of C 301874434

scaffold %G 22.38 number of G 301795420

scaffold %T 27.62 number of T 372383187

scaffold %N 0.00 number of N 17800

scaffold %non-ACGTN 0.00

Number of scaffold non-ACGTN nt 0

Percentage of assembly in scaffolded contigs 89.1%

Percentage of assembly in unscaffolded contigs 10.9%

Average number of contigs per scaffold 1.1

Average length of break (>=10 N) between contigs in scaffold 100

Number of contigs 1365

Number of contigs in scaffolds 237

Number of contigs not in scaffolds 1128

Total size of contigs 1348393292

Longest contig 100012138

Shortest contig 520

Number of contigs > 1K nt 1342 98.3%

Number of contigs > 10K nt 1118 81.9%

Number of contigs > 100K nt 436 31.9%

Number of contigs > 1M nt 86 6.3%

Number of contigs > 10M nt 30 2.2%

Mean contig size 987834

Median contig size 48791

N50 contig length 37118489 L50 contig count 12

N60 contig length 22365136 L60 contig count 17

N70 contig length 18196450 L70 contig count 24

N80 contig length 8000318 L80 contig count 35

N90 contig length 1303612 L90 contig count 75

contig %A 27.61 number of A 372340251

contig %C 22.39 number of C 301874434

**purge_dups assembly statistics**

**Table S7** - Assembly statistics from the purge-dupped assembly

---------------- Information for assembly 'assembly_v0.B.fasta' ----------------

Number of scaffolds 476

Total size of scaffolds 1310822856

Longest scaffold 218127570

Shortest scaffold 520

Number of scaffolds > 1K nt 471 98.9%

Number of scaffolds > 10K nt 419 88.0%

Number of scaffolds > 100K nt 247 51.9%

Number of scaffolds > 1M nt 41 8.6%

Number of scaffolds > 10M nt 18 3.8%

Mean scaffold size 2753830

Median scaffold size 106586

N50 scaffold length 84761895 L50 scaffold count 5

N60 scaffold length 72296394 L60 scaffold count 7

N70 scaffold length 55825834 L70 scaffold count 9

N80 scaffold length 24958351 L80 scaffold count 12

N90 scaffold length 8122458 L90 scaffold count 21

scaffold %A 27.87 number of A 365279929

scaffold %C 22.17 number of C 290569076

scaffold %G 22.14 number of G 290210744

scaffold %T 27.83 number of T 364747907

scaffold %N 0.00 number of N 15200

scaffold %non-ACGTN 0.00

Number of scaffold non-ACGTN nt 0

Percentage of assembly in scaffolded contigs 90.7%

Percentage of assembly in unscaffolded contigs 9.3%

Average number of contigs per scaffold 1.3

Average length of break (>=10 N) between contigs in scaffold 100

Number of contigs 628

Number of contigs in scaffolds 193

Number of contigs not in scaffolds 435

Total size of contigs 1310807656

Longest contig 100012138

Shortest contig 1

Number of contigs > 1K nt 618 98.4%

Number of contigs > 10K nt 557 88.7%

Number of contigs > 100K nt 337 53.7%

Number of contigs > 1M nt 85 13.5%

Number of contigs > 10M nt 30 4.8%

Mean contig size 2087273

Median contig size 118225

N50 contig length 37118489 L50 contig count 12

N60 contig length 22569012 L60 contig count 16

N70 contig length 18619766 L70 contig count 22

N80 contig length 9622368 L80 contig count 32

N90 contig length 2424725 L90 contig count 56

contig %A 27.87 number of A 365279929

contig %C 22.17 number of C 290569076

**Gap filled assembly statistics**

**Table S8** - Assembly statistics from the gap filled assembly

Total size of scaffolds 1311889610

Longest scaffold 218158159

Shortest scaffold 520

Number of scaffolds > 1K nt 471 98.9%

Number of scaffolds > 10K nt 419 88.0%

Number of scaffolds > 100K nt 247 51.9%

Number of scaffolds > 1M nt 41 8.6%

Number of scaffolds > 10M nt 18 3.8%

Mean scaffold size 2756071

Median scaffold size 106586

N50 scaffold length 85146451 L50 scaffold count 5

N60 scaffold length 72297643 L60 scaffold count 7

N70 scaffold length 55826375 L70 scaffold count 9

N80 scaffold length 25011129 L80 scaffold count 12

N90 scaffold length 8151782 L90 scaffold count 21

scaffold %A 27.86 number of A 365551176

scaffold %C 22.17 number of C 290832808

scaffold %G 22.14 number of G 290482487

scaffold %T 27.82 number of T 365014839

scaffold %N 0.00 number of N 8300

scaffold %non-ACGTN 0.00

Number of scaffold non-ACGTN nt 0

Percentage of assembly in scaffolded contigs 88.4%

Percentage of assembly in unscaffolded contigs 11.6%

Average number of contigs per scaffold 1.2

Average length of break (>=10 N) between contigs in scaffold 100

Number of contigs 559

Number of contigs in scaffolds 111

Number of contigs not in scaffolds 448

Total size of contigs 1311881310

Longest contig 140716282

Shortest contig 520

Number of contigs > 1K nt 550 98.4%

Number of contigs > 10K nt 491 87.8%

Number of contigs > 100K nt 300 53.7%

Number of contigs > 1M nt 70 12.5%

Number of contigs > 10M nt 27 4.8%

Mean contig size 2346836

Median contig size 113493

N50 contig length 42764735 L50 contig count 9

N60 contig length 35788093 L60 contig count 12

N70 contig length 22253039 L70 contig count 17

N80 contig length 15526072 L80 contig count 24

N90 contig length 3254054 L90 contig count 41

contig %A 27.86 number of A 365551176

contig %C 22.17 number of C 290832808

contig %G 22.14 number of G 290482487

contig %T 27.82 number of T 365014839

contig %N 0.00 number of N 0

contig %non-ACGTN 0.00

Number of contig non-ACGTN nt 0

**Final assembly statistics**

**Table S9** - Final assembly statistics using the flye assembly.

Total size of scaffolds 1311830680

Longest scaffold 218158159

Shortest scaffold 5019

Number of scaffolds > 1K nt 450 100.0%

Number of scaffolds > 10K nt 419 93.1%

Number of scaffolds > 100K nt 247 54.9%

Number of scaffolds > 1M nt 41 9.1%

Number of scaffolds > 10M nt 18 4.0%

Mean scaffold size 2915179

Median scaffold size 119830

N50 scaffold length 85146451 L50 scaffold count 5

N60 scaffold length 72297643 L60 scaffold count 7

N70 scaffold length 55826375 L70 scaffold count 9

N80 scaffold length 25011129 L80 scaffold count 12

N90 scaffold length 8151782 L90 scaffold count 21

scaffold %A 27.86 number of A 365536410

scaffold %C 22.17 number of C 290818306

scaffold %G 22.14 number of G 290467092

scaffold %T 27.82 number of T 365000572

scaffold %N 0.00 number of N 8300

scaffold %non-ACGTN 0.00

Number of scaffold non-ACGTN nt 0

Percentage of assembly in scaffolded contigs 88.4%

Percentage of assembly in unscaffolded contigs 11.6%

Average number of contigs per scaffold 1.2

Average length of break (>=10 N) between contigs in scaffold 100

Number of contigs 533

Number of contigs in scaffolds 111

Number of contigs not in scaffolds 422

Total size of contigs 1311822380

Longest contig 140716282

Shortest contig 578

Number of contigs > 1K nt 529 99.2%

Number of contigs > 10K nt 491 92.1%

Number of contigs > 100K nt 300 56.3%

Number of contigs > 1M nt 70 13.1%

Number of contigs > 10M nt 27 5.1%

Mean contig size 2461205

Median contig size 129703

N50 contig length 42764735 L50 contig count 9

N60 contig length 35788093 L60 contig count 12

N70 contig length 22253039 L70 contig count 17

N80 contig length 15526072 L80 contig count 24

N90 contig length 3254054 L90 contig count 41

contig %A 27.86 number of A 365536410

contig %C 22.17 number of C 290818306

contig %G 22.14 number of G 290467092

contig %T 27.82 number of T 365000572

contig %N 0.00 number of N 0

contig %non-ACGTN 0.00

Number of contig non-ACGTN nt 0

**Table S10** - BUSCO results from the final assembly

bLeucoFulig_1.0_cpa1_aves/full_table_w_busco_completes.tsv

aves_odb10 n:8338

8334 Complete BUSCOs (C) 99.95%

8317 Complete and single-copy BUSCOs (S) 99.75%

17 Complete and duplicated BUSCOs (D) 0.20%

3 Fragmented BUSCOs (F) 0.04%

1 Missing BUSCOs (M) 0.01%

8338 Total BUSCO aves_odb10 group 100.00%

**Table S11** - Final assembly statistics with the BUSCO results for pseudochromosomes.

Chr1_Lful 218158159 218,158,159 16.63% 1 B:1257 C:1254 F:1 D:1 d:2 telomeres: TOP

Chr2_Lful 168060181 386,218,340 29.44% 2 B:767 C:766 F:0 D:1 d:1

Chr3_Lful 127072349 513,290,689 39.13% 3 B:736 C:736 F:0 D:0 d:0

Chr4_Lful 94676801 607,967,490 46.34% 4 B:780 C:780 F:0 D:0 d:0

ChrZ_Lful 85146451 693,113,941 52.83% 5 B:374 C:363 F:0 D:11 d:11 telomeres: BOTTOM

Chr5_Lful 84295662 777,409,603 59.26% 6 B:449 C:449 F:0 D:0 d:0

Chr6_Lful 72297643 849,707,246 64.77% 7 B:592 C:589 F:1 D:1 d:2

Chr7_Lful 57391743 907,098,989 69.15% 8 B:497 C:496 F:1 D:0 d:0

Chr8_Lful 55826375 962,925,364 73.40% 9 B:559 C:559 F:0 D:0 d:0

Chr9_Lful 49124529 1,012,049,893 77.15% 10 B:421 C:419 F:0 D:1 d:2

ChrW_Lful 25640768 1,037,690,661 79.10% 11 B:12 C:0 F:0 D:12 d:12 *

Chr10_Lful 25011129 1,062,701,790 81.01% 12 B:195 C:195 F:0 D:0 d:0

Chr11_Lful 22317082 1,085,018,872 82.71% 13 B:179 C:178 F:0 D:1 d:1

Chr12_Lful 18121858 1,103,140,730 84.09% 14 B:186 C:186 F:0 D:0 d:0

Chr13_Lful 17257219 1,120,397,949 85.41% 15 B:232 C:232 F:0 D:0 d:0 telomeres: BOTTOM

Chr14_Lful 15526072 1,135,924,021 86.59% 16 B:171 C:171 F:0 D:0 d:0

Chr15_Lful 13248692 1,149,172,713 87.60% 17 B:177 C:177 F:0 D:0 d:0 telomeres: TOP

Chr16_Lful 11705882 1,160,878,595 88.49% 18 B:138 C:138 F:0 D:0 d:0

Chr17_Lful 9460977 1,170,339,572 89.21% 19 B:101 C:99 F:0 D:1 d:2 telomeres: BOTTOM

Chr19_Lful 8717267 1,179,056,839 89.88% 20 B:115 C:115 F:0 D:0 d:0 telomeres: TOP

Chr18_Lful 8151782 1,187,208,621 90.50% 21 B:87 C:87 F:0 D:0 d:0

Chr20_Lful 8000318 1,195,208,939 91.11% 22 B:124 C:124 F:0 D:0 d:0 telomeres: BOTTOM

Chr21_Lful 7573479 1,202,782,418 91.69% 23 B:105 C:105 F:0 D:0 d:0

Chr22_Lful 6903002 1,209,685,420 92.21% 24 B:47 C:47 F:0 D:0 d:0

Chr23_Lful 4067773 1,213,753,193 92.52% 25 B:28 C:28 F:0 D:0 d:0 telomeres: BOTTOM

Chr24_Lful 3465058 1,217,218,251 92.79% 26 B:21 C:20 F:0 D:1 d:1

contig_1250 2714326 1,219,932,577 92.99% 27

contig_3210 2424725 1,222,357,302 93.18% 28

Chr28_Lful 2194621 1,224,551,923 93.35% 29

Chr29_Lful 2167830 1,226,719,753 93.51% 30 B:1 C:1 F:0 D:0 d:0

Chr25_Lful 2107369 1,228,827,122 93.67% 31

Chr26_Lful 2077667 1,230,904,789 93.83% 32 B:3 C:3 F:0 D:0 d:0

contig_2371 2010826 1,232,915,615 93.98% 33

Chr32_Lful 1724086 1,234,639,701 94.11% 34

Chr27_Lful 1664944 1,236,304,645 94.24% 35

Chr30_Lful 1663535 1,237,968,180 94.37% 36 telomeres: BOTTOM

Chr31_Lful 1401952 1,239,370,132 94.48% 37

**Repeats Breakdown**

**RepeatMasker Table**

**Table S12** - Repeatmasker breakdown of number and type of repeats in the assembly.

==================================================

file name: lavagull_v0.C.fasta

sequences: 476

total length: 1311889610 bp (1311881310 bp excl N/X-runs)

GC level: 44.31 %

bases masked: 208510090 bp ( 15.89 %)

==================================================

number of length percentage

elements* occupied of sequence

--------------------------------------------------

Retroelements 222300 100360677 bp 7.65 %

SINEs: 12950 1655322 bp 0.13 %

Penelope: 178 36812 bp 0.00 %

LINEs: 173890 74360715 bp 5.67 %

CRE/SLACS 0 0 bp 0.00 %

L2/CR1/Rex 164747 72998486 bp 5.56 %

R1/LOA/Jockey 262 247672 bp 0.02 %

R2/R4/NeSL 6270 399811 bp 0.03 %

RTE/Bov-B 239 154336 bp 0.01 %

L1/CIN4 94 23933 bp 0.00 %

LTR elements: 35460 24344640 bp 1.86 %

BEL/Pao 0 0 bp 0.00 %

Ty1/Copia 0 0 bp 0.00 %

Gypsy/DIRS1 1968 665320 bp 0.05 %

Retroviral 33372 23661210 bp 1.80 %

DNA transposons 48287 7360146 bp 0.56 %

hobo-Activator 7044 1163111 bp 0.09 %

Tc1-IS630-Pogo 850 147611 bp 0.01 %

En-Spm 0 0 bp 0.00 %

MULE-MuDR 0 0 bp 0.00 %

PiggyBac 0 0 bp 0.00 %

Tourist/Harbinger 18433 2026019 bp 0.15 %

Other (Mirage, 0 0 bp 0.00 %

P-element, Transib)

Rolling-circles 2733 437748 bp 0.03 %

Unclassified: 33767 10673912 bp 0.81 %

Total interspersed repeats: 118431547 bp 9.03 %

Small RNA: 3471 517329 bp 0.04 %

Satellites: 21800 75199385 bp 5.73 %

Simple repeats: 264861 11206023 bp 0.85 %

Low complexity: 58652 3109769 bp 0.24 %

==================================================

RepeatMasker version 4.1.7-p1 , sensitive mode

run with rmblastn version 2.14.0+

The query was compared to classified sequences in ".../denovo_and_aves_curated_repeats_Dfam38.fa"

**Table S13** - Per chromosome account of length of repeats and percentage.

# Name Record length Non-repeat len Repeat length Repeat % Cumulative %

Chr1_Lful 218158159 191748155 26410004 12.11% 12.11%

Chr2_Lful 168060181 148933533 19126648 11.38% 11.79%

Chr3_Lful 127072349 113451202 13621147 10.72% 11.53%

Chr4_Lful 94676801 86991682 7685119 8.12% 10.99%

Chr5_Lful 84295662 75621140 8674522 10.29% 10.91%

Chr6_Lful 72297643 66979194 5318449 7.36% 10.57%

Chr7_Lful 57391743 53048968 4342775 7.57% 10.36%

Chr8_Lful 55826375 51320794 4505581 8.07% 10.22%

Chr9_Lful 49124529 45484184 3640345 7.41% 10.07%

Chr10_Lful 25011129 23390813 1620316 6.48% 9.97%

Chr11_Lful 22317082 20673870 1643212 7.36% 9.91%

Chr12_Lful 18121858 16720429 1401429 7.73% 9.87%

Chr13_Lful 17257219 16065213 1192006 6.91% 9.82%

Chr14_Lful 15526072 13498356 2027716 13.06% 9.87%

Chr15_Lful 13248692 12342886 905806 6.84% 9.83%

Chr16_Lful 11705882 9143868 2562014 21.89% 9.97%

Chr17_Lful 9460977 7985467 1475510 15.60% 10.02%

Chr18_Lful 8151782 6680513 1471269 18.05% 10.08%

Chr19_Lful 8717267 7921765 795502 9.13% 10.07%

Chr20_Lful 8000318 7308780 691538 8.64% 10.06%

Chr21_Lful 7573479 6052324 1521155 20.09% 10.13%

Chr22_Lful 6903002 5978499 924503 13.39% 10.15%

Chr23_Lful 4067773 3361239 706534 17.37% 10.18%

Chr24_Lful 3465058 2991569 473489 13.66% 10.19%

Chr25_Lful 2107369 1719362 388007 18.41% 10.20%

Chr26_Lful 2077667 1360878 716789 34.50% 10.25%

Chr27_Lful 1664944 1021463 643481 38.65% 10.29%

Chr28_Lful 2194621 1637901 556720 25.37% 10.32%

Chr29_Lful 2167830 1633801 534029 24.63% 10.35%

Chr30_Lful 1663535 1350508 313027 18.82% 10.36%

Chr31_Lful 1401952 1088483 313469 22.36% 10.38%

Chr32_Lful 1724086 1350382 373704 21.68% 10.40%

ChrW_Lful 25640768 10473681 15167087 59.15% 11.49%

ChrZ_Lful 85146451 74106416 11040035 12.97% 11.59%

**Genome Annotation**

**Table S14** - Comparison of the expected genome length, unique length, and repeat length from Genomescope2 and that for the final assembly.

|  | Genomescope2 Bp | Pct of Genome Length | Assembly | Pct of Assembly | Bp Diff | Pct Diff |
| --- | --- | --- | --- | --- | --- | --- |
| Genome Length | 1,167,341,962 | 100% | 1,311,830,680 | 100.00% | -144,488,718 | -11.01% |
| Genome Unique Length | 1,083,979,602 | 92.86% | 1,103,325,722 | 84.11% | -19,346,120 | -1.75% |
| Genome Repeat Length | 83,362,360 | 7.14% | 208,504,958 | 15.89% | -125,142,598 | -60.02% |

**Table S15** - Functional annotation standard gene model basic statistics

| **Annotation type** | **Value** |
| --- | --- |
| Number of genes | 21,808 |
| Genome percentage | 17.34% |
| Number of mRNA | 23,928 |
| mRNA mean length | 11,216.08 bp |
| Mean exons per mRNA | 7.89 |
| Mean mRNA exon len. | 173.24 |
| Single exon genes | 3,587 |
| Named genes | 20,027 |
| Named mRNA | 22,139 |
| BUSCO aves_odb10 lineage | 96.56% / 8051 |

**Table S16** - Comparison of the number of genes, mRNAs, genes with names, and mRNAs with names found from different functional annotation gene sets (candidate, standard, and protein)

|  | Genes | mRNA | Genes with Names | mRNAs with Names |
| --- | --- | --- | --- | --- |
| Candidate Set | 24,768 | 26,902 | 20,027 | 22,139 |
| Quality Filtered Standard Set | 21,808 | 23,928 | 20,027 | 22,139 |
| Protein Domains, One of More | 16,432 | 18,478 | 16,407 | 18,453 |
|  | *Removes 21 nested genes and 25 mRNAs without a start or stop codon from BRAKER output gff |  |  |  |

**Table S17** - OMArk statistics

| **OMArk statistic** | **Value** |
| --- | --- |
| Consistent | 17954 (82.33%) |
| Inconsistent | 2,472 (11.34%) |
| Unknown | 1,382 (6.34%) |

**Table S18** - Annotation results for each of the 37 reads of the mitogenome

gh F rrnS V rrnL L2 I Q M W A N C Y S2 D K G R H S1 L1 T P E gh F rrnS V rrnL L2 I Q M W A N C Y S2 D K G R H S1 L1 T P E

31fac7cb-7839-41d3-bd41-e6b626a31a59_RC F OH rrnS V rrnL L2 I Q M

45f14f55-2bb4-4d11-adab-bf18f370e436 F OH rrnS V rrnL L2 I Q M W A N C Y S2 D K

4d4695fc-1fb5-4265-a335-64b35bcc5b52_RC F OH rrnS V rrnL L2 I Q M W A N C Y S2 D K G

5d75ce4a-80e3-4f2d-a6c3-290b9104595e_RC F OH rrnS V rrnL L2 I Q M W A N C Y S2 D K G R H S1 L1 T P E gh gh

669d7c95-871a-43cf-bb58-f2c647ef5086_RC F OH rrnS V rrnL L2 I Q M W A N C Y S2 D K G R

7a7588d5-5c3b-42ab-afbf-b5a862142676 F OH rrnS V rrnL L2 I Q M

80dcfbeb-7267-4e18-ba2b-e868e1ee49bd_RC F OH rrnS V rrnL L2 I Q M W A N C Y S2 D K G R H S1 L1

88da352d-bf5a-481c-b06c-b565b88b435a_RC F OH rrnS V rrnL L2 I Q M W A N C Y S2 D K G R H S1 L1 T P

90f308f8-accb-4b55-a24b-01b3a8dac591_RC F OH rrnS V rrnL L2 I Q M W A N C Y S2 D K G R H S1 L1

70472abe-5b90-4e93-891f-aa6153bf6397 rrnS V rrnL L2 I Q M W A N C Y S2 D K G R H S1 L1 T P E gh gh OH T P # T template mismatch

96e25135-5f77-4892-b541-0cd4245fd6c9:0_RC rrnS OH V rrnL L2 I Q M

e310a34e-4ffc-4d4c-bf36-459b60bcf368_RC rrnS OH V rrnL L2 I Q M W A N C Y S2 D

4a99a8f9-a245-4f10-bdd0-ed26cc72ef39:1 rrnL L2 I Q M W A N C Y S2 D

8a2dec04-74ab-4e80-8162-22ce14f0ee58 rrnL L2 I Q M W A N C Y S2 D K G R H S1 L1 T P

b398e253-cd1f-43c4-b052-7bb0d05347ea rrnL L2 I Q M W A N C Y S2 D K G R

cc537c70-4c5a-480c-862c-9d99ea885e7a rrnL L2 I Q M W A N C Y S2 D K G R H S1 L1 T P E gh gh T P E OH # T template mismatch

f515d4bd-6afd-4be5-9bf8-17426b0300cb_RC rrnL L2 I Q M W A N C Y S2 D K G R

f715c6d7-ab5e-4f52-8131-3cc3e449a71a_RC rrnL L2 I Q M W A N C Y S2 D K G R H S1 L1 T P E gh gh OH

8d4828f6-ab30-4d09-abd1-9fbb75260d7d I Q M W A N C Y

96e25135-5f77-4892-b541-0cd4245fd6c9:1_RC W A N C Y S2 D K G R H S1 L1 OH T P E

8f4967ac-12c1-4512-b757-24be1dd903b2_RC S2 D K G R H S1 L1

ef38988d-9a8b-4eea-a10c-ac4b537c3530 S2 D K G R H S1 L1 T P E gh gh OH T P E # T template mismatch

23fa5033-a8e7-4e1e-9b97-3c42313f89d5_RC K G R H S1 L1

52a39b43-08c9-4daf-9767-9c92dbced424 G R H S1 L1 T P E gh gh OH

e8591260-df3f-4a6a-bca0-7e1a9ce072d0 G R H S1 L1 T

1657b6a2-caee-487d-80d7-c782c32ada07 H S1 L1 T P E gh gh T P E OH # T template mismatch

ba34c200-0fad-441d-acb8-08e13ed2d0b1 H S1 L1 T P E gh gh OH

3e0ac441-2dfb-4b25-9863-28153f57fdd5 T P E gh OH F rrnS V rrnL L2 I Q M W A N C Y S2 D K G R H S1 L1

4a99a8f9-a245-4f10-bdd0-ed26cc72ef39:0 T P E gh gh OH T P # T template mismatch

8285d898-7e18-4af2-9830-e86f8f2f2d75 T P E gh OH F rrnS V rrnL L2

f19ae507-68c2-497c-86fa-af4cdfd34a5e T P E gh gh OH T P E gh # T template mismatch

f98eade5-e837-48e3-90c5-b1d928676661 T P E gh OH F rrnS V rrnL L2 I Q M W A N C Y S2 D K G R H S1 L1

49d43429-4327-46c7-b696-b867907f6c68 E gh T P E gh OH F rrnS V rrnL L2 I Q M W A N C Y S2 D K G R H S1 L1 # T template mismatch

4a3234d9-f5d6-40b9-a68e-40c904df0b1b:0 OH T P E # N too few items

6a1c371b-b593-48f2-a498-5ceeaa24e7ca_RC OH F rrnS V rrnL L2 I Q M

9e1725c4-b7d5-496a-9951-241bbfc2a96e_RC OH F rrnS V rrnL L2 I Q M W A N C Y S2 D K G R H S1 L1 T P

c79ede3d-f646-4725-bab4-f230a881503b_RC OH rrnS V rrnL L2 I Q M W A N C Y S2 D K G R H S1 L1 T P E gh gh

**Missing BUSCOs**

**Table S19** - Missing BUSCOs for the lava gull, lesser black-backed gull, yellow-legged gull, black-headed gull, laughing gull, and European herring gull.

Lava gull

28097at8782 Missing <https://v10-1.orthodb.org/?query=28097at8782>

Lesser black-backed gull

28097at8782 Missing <https://v10-1.orthodb.org/?query=28097at8782>

Yellow-legged gull

28097at8782 Missing <https://v10-1.orthodb.org/?query=28097at8782>

38263at8782 Missing

6746at8782 Missing

Black-headed gull

28097at8782 Missing <https://v10-1.orthodb.org/?query=28097at8782>

50276at8782 Missing

Laughing gull (missing 528 other BUSCOs)

28097at8782 Missing <https://v10-1.orthodb.org/?query=28097at8782>

European herring gull

19299at8782 Missing

27421at8782 Missing

28097at8782 Missing <https://v10-1.orthodb.org/?query=28097at8782>

50276at8782 Missing

**Middle telomeres in lesser black-backed gull Chromosome Z**

**Table S20** - Middle telomeres located within the Z chromosome of the lesser black-backed gull.

LfusZ MIDDLE 213823347 25308bp 88144435..88169742 41.223%

LfusZ MIDDLE 213823347 3552bp 92800219..92803770 43.400%

LfusZ MIDDLE 213823347 1998bp 93180727..93182724 43.578%

LfusZ MIDDLE 213823347 4662bp 93182947..93187608 43.579%

LfusZ MIDDLE 213823347 5106bp 98860819..98865924 46.235%

LfusZ MIDDLE 213823347 2442bp 111389389..111391830 52.094%

LfusZ MIDDLE 213823347 10656bp 119858911..119869566 56.055%

LfusZ MIDDLE 213823347 7548bp 120382831..120390378 56.300%

LfusZ MIDDLE 213823347 1776bp 127272379..127274154 59.522%

LfusZ MIDDLE 213823347 2886bp 134569297..134572182 62.935%

LfusZ MIDDLE 213823347 5772bp 135048595..135054366 63.159%

LfusZ MIDDLE 213823347 3108bp 141189559..141192666 66.031%

LfusZ MIDDLE 213823347 1776bp 144353281..144355056 67.511%

LfusZ MIDDLE 213823347 1332bp 147046363..147047694 68.770%

LfusZ MIDDLE 213823347 1554bp 155570497..155572050 72.757%

LfusZ MIDDLE 213823347 2664bp 155572273..155574936 72.757%

LfusZ MIDDLE 213823347 6438bp 156331069..156337506 73.112%

LfusZ MIDDLE 213823347 3330bp 156451615..156454944 73.169%

LfusZ MIDDLE 213823347 1776bp 156918481..156920256 73.387%

LfusZ MIDDLE 213823347 12432bp 165999835..166012266 77.634%

LfusZ MIDDLE 213823347 1554bp 166763515..166765068 77.991%

LfusZ MIDDLE 213823347 1776bp 168373459..168375234 78.744%

LfusZ MIDDLE 213823347 9546bp 194560357..194569902 90.991%

LfusZ MIDDLE 213823347 5994bp 202712197..202718190 94.804%

LfusZ MIDDLE 213823347 2664bp 207350665..207353328 96.973%

LfusZ MIDDLE 213823347 5550bp 208035979..208041528 97.293%


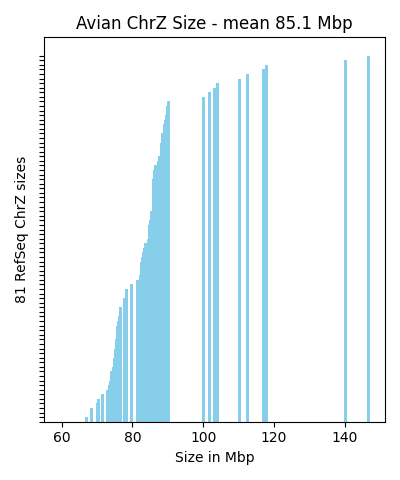


**Figure S1** - Bar chart of Z chromosome length in 81 RefSeq avian genomes.

| 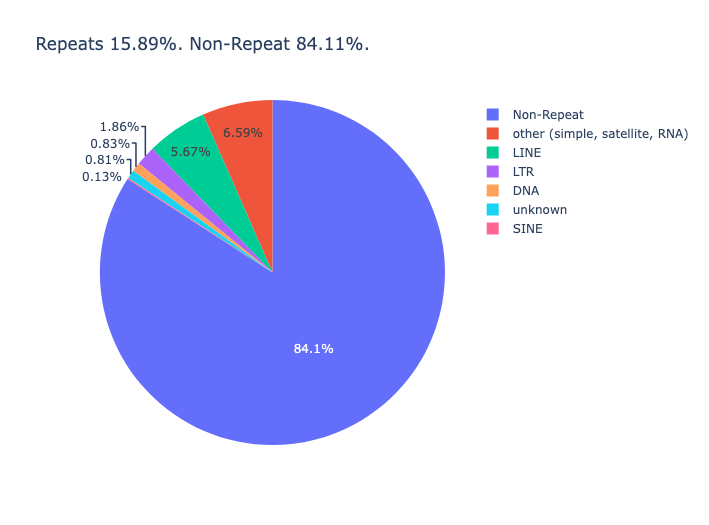 |
| --- |

**Figure S2** - Classification of repeats from RepeatMasker. The lava gull genome is 15.89% repeats and 84.11% non-repeat sequence.

**
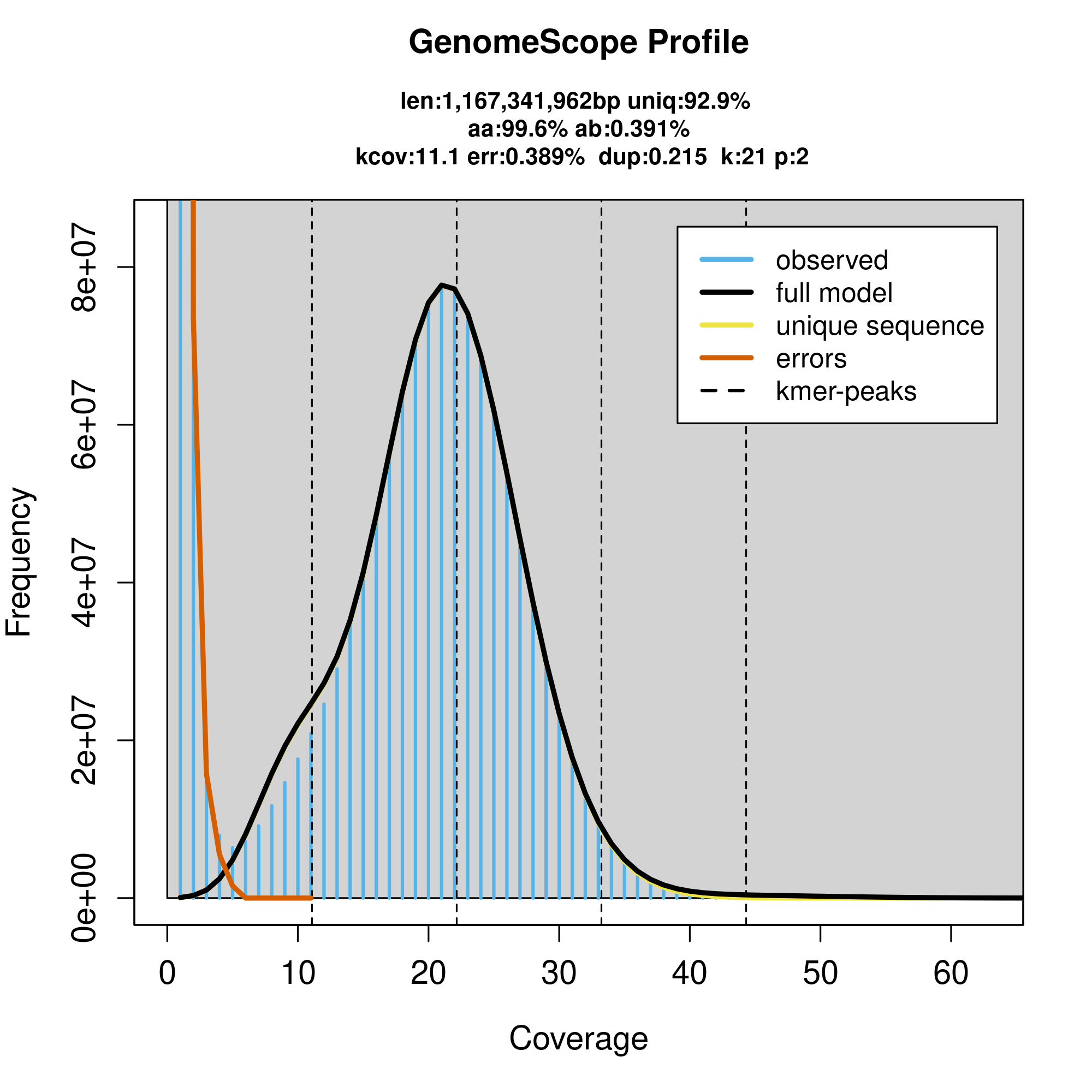
**

**Figure S3** - GenomeScope plots using kmer length 21.


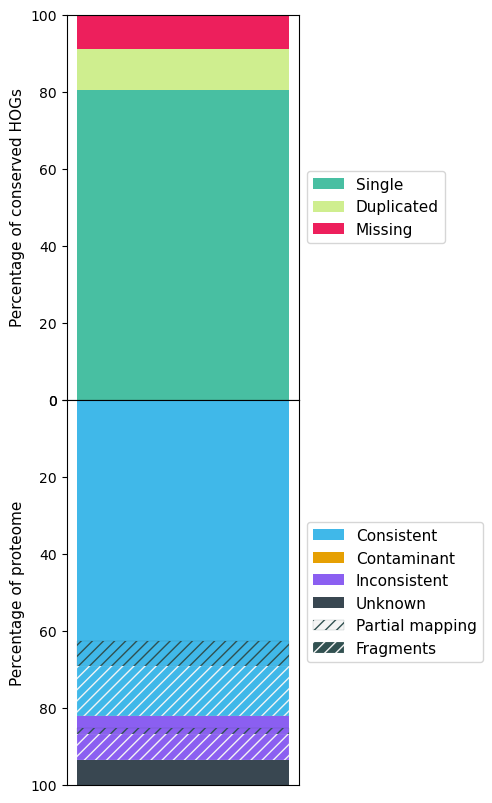


**Figure S4** - OMArk HOGs analysis chart
